# AltHapAlignR: improved accuracy of RNA-seq analyses through the use of alternative haplotypes

**DOI:** 10.1093/bioinformatics/bty125

**Published:** 2018-03-05

**Authors:** Wanseon Lee, Katharine Plant, Peter Humburg, Julian C Knight

**Affiliations:** Wellcome Centre for Human Genetics, University of Oxford, Oxford, UK

## Abstract

**Motivation:**

Reliance on mapping to a single reference haplotype currently limits accurate estimation of allele or haplotype-specific expression using RNA-sequencing, notably in highly polymorphic regions such as the major histocompatibility complex.

**Results:**

We present AltHapAlignR, a method incorporating alternate reference haplotypes to generate gene- and haplotype-level estimates of transcript abundance for any genomic region where such information is available. We validate using simulated and experimental data to quantify input allelic ratios for major histocompatibility complex haplotypes, demonstrating significantly improved correlation with ground truth estimates of gene counts compared to standard single reference mapping. We apply AltHapAlignR to RNA-seq data from 462 individuals, showing how significant underestimation of expression of the majority of classical human leukocyte antigen genes using conventional mapping can be corrected using AltHapAlignR to allow more accurate quantification of gene expression for individual alleles and haplotypes.

**Availability and implementation:**

Source code freely available at https://github.com/jknightlab/AltHapAlignR.

**Supplementary information:**

Supplementary data are available at *Bioinformatics* online.

## 1 Introduction

RNA sequencing (RNA-seq) enables high resolution quantification of transcription (Wang *et al.*, 2009). There are, however, significant opportunities for bias in transcript quantification estimates in favor of reference alleles (Degner *et al.*, 2009) dependent, for example, on the particular sequencing technology used and length of read generated, the strategy and reference sequence adopted for mapping and the processing pipeline (Conesa *et al.*, 2016; Reinert *et al.*, 2015). The development of longer read technologies for high-throughput sequencing will help address this problem but given the large amounts of data that are already generated and will continue to be generated, using shorter reads there is a need for innovative approaches to enable more accurate quantification. This is particularly the case for highly polymorphic regions of the genome where gene and transcript level expression data may be of significant clinical and biological interest such as the major histocompatibility complex (MHC) (Brandt *et al.*, 2015; Lighten *et al.*, 2014). Quantification of allele- or haplotype-specific gene expression is of particular interest when considering the functional significance of regulatory genetic variants such as arising through genome-wide association studies of common disease, where such variants are most commonly implicated (Schaub *et al.*, 2012). Establishing causal mechanistic relationships between specific variants and expression of individual genes is a current priority in this field of research and accurate quantification of transcription is a critical step in such studies (Knight, 2014).

The MHC highlights some of the challenges in current application of RNA-seq. The MHC is extremely polymorphic and gene dense, with an essential role for classical human leukocyte antigens (HLA) in transplantation, established associations with disease, notably autoimmunity and infection, as well as adverse drug reactions (Matzaraki *et al.*, 2017). It is proposed that a number of these trait associations involve effects on gene expression, but the definition of functional alleles, such as through gene expression quantitative trait mapping, has been limited by the inability to obtain accurate quantification of transcript abundance. The need to define such variants, as well as broader clinical imperatives to apply NGS (next-generation sequencing) accurately to the MHC for tissue typing, have driven efforts to address accurate read mapping for the MHC (Carapito *et al.*, 2016). The high degree of diversity in the MHC and occurrence of close paralogues for several genes causes major issues for standard mapping methods (Brandt, *et al.*, 2015). Methods that utilize *de novo* assembly with genome inference have been developed and applied to establish personal reference sequences (Chaisson *et al.*, 2015; Dilthey *et al.*, 2015). Other methods rely on the availability of genotyping data to support the polymorphism-aware mapping of RNA-seq reads (Baker *et al*., 2015; Pandey *et al.*, 2013; Rozowsky *et al.*, 2011; Sun, 2012; Turro *et al.*, 2011). Some methods, like MMSEQ (Turro *et al.*, 2011), QuASAR (Harvey *et al.*, 2015) and Salmon (Patro *et al.*, 2017), can obtain allele-specific expression estimates using only RNA-seq data, although QuASAR is limited to a set of known polymorphisms and MMSEQ only allows use of a single haplotype reference. There is also the opportunity in the MHC to leverage the availability of sequences for eight of the most commonly occurring long range (extended) haplotypes in Caucasian populations that have been generated for the MHC to use as alternative reference haplotypes (Horton *et al.*, 2008). Here, we show how such information can be used in a novel strategy for more accurate RNA-seq analysis that leverages knowledge of haplotype sequence and structure. The MHC is presented as an exemplar, but the methodology is generic and applicable to other genomic regions for which alternate reference sequences are increasingly available (Schneider *et al.*, 2017).

## 2 Materials and methods

### 2.1 Pipeline overview

**Fig. 1.**
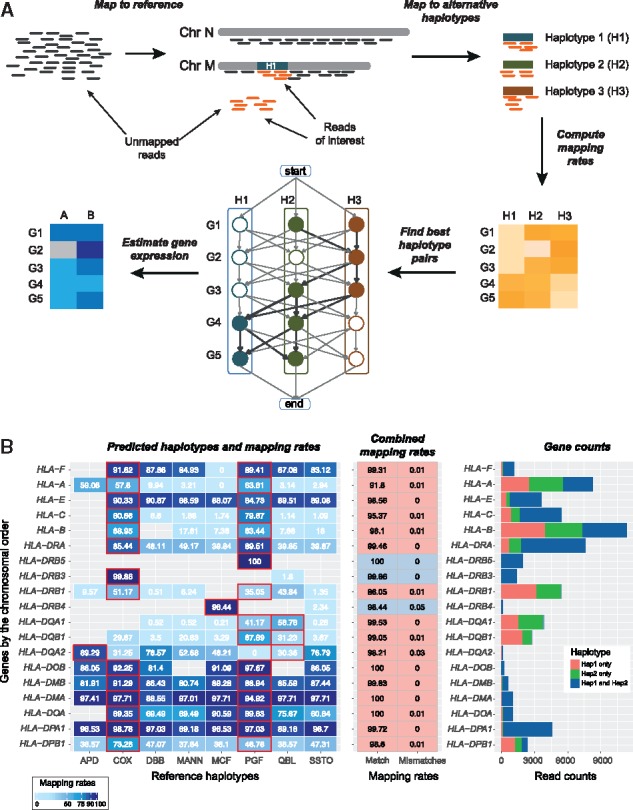
Overview of AltHapAlignR workflow and sample output with haplotype prediction. (**A**) Schematic depicting pipeline. Preprocessing includes mapping of reads to the reference haplotype with unmapped reads extracted and realigned to the alternative haplotypes independently. Using the scoring weights of the haplotype pairs (based on editing distances of aligned reads to the respective references), closest haplotypes are selected for each gene while maximizing the quality of read alignments and minimizing the number of switches between haplotypes. Gene and haplotype level expression is estimated based on reads aligned to the selected haplotypes. (**B**) Example of AltHapAlignR output using synthetic heterozygote data (PGF and COX 1:1 ratio). *Haplotype prediction and mapping rates* (*left panel*). Illustrated for each classical HLA gene (y-axis) and eight known haplotypes. Numbers in each cell are mapping rates in each haplotype. Predicted haplotypes highlighted with red border. Empty cells represent genes not annotated in given haplotype. *Combined mapping rates from the predicted haplotypes* (*middle panel).* Mapping rate (first column): read counts of gene in the predicted haplotype(s)/total read count of the gene across all haplotypes. Mismatch mapping rates of predicted haplotypes (second column). Pink (predicted heterozygote), grey (homozygotes). *Gene counts* (r*ight panel*). Raw read counts for each HLA gene

The AltHapAlignR pipeline consists of three main stages (Fig. 1).

**Stage 1.** Reads are aligned to the standard genome reference, which currently comprises the PGF haplotype sequence for the MHC region as part of the Genome Reference Consortium human genome (build 38) (GRCh38). Reads mapped to the MHC (chr6: 28 520 000–33 390 000, GRCh38) and unmapped reads are extracted and realigned to the other MHC reference haplotypes independently. Reads mapped to multiple regions, with mapping quality less than 20, and duplicate reads, are removed. Any third party read mapper, like TopHat (Kim *et al.*, 2013) or HISAT2 (Kim *et al.*, 2015), can be used for this purpose, provided that information about the alignment is stored in the standard tags provided by the SAM format specification.

**Stage 2.** AltHapAlignR estimates expression of genes and haplotype using alignments to the available reference haplotypes, here the eight MHC Haplotype Project reference sequences. Each read is assigned to a gene or genes according to reference annotation. For paired-end reads, both reads in a pair must align to the same gene. Reads assigned to multiple genes or to different genes on different haplotypes are removed. For each gene, all reads mapped to at least one of the reference haplotypes are weighted based on the editing distances (number of substitutions, insertions and deletions) between the reads and reference sequences. For paired-end reads, editing distances for both reads are combined. The resulting weights across all eight haplotypes are normalized to 1. For each gene/haplotype pair, the mapping rate is calculated as the sum of weighted values divided by the total number of reads mapped to this gene across the eight haplotypes. To infer the best pair of reference haplotypes, we obtained combined mapping rates and editing distances of all possible haplotype pairs. For a given gene, we used the haplotype pairs that produce the lowest relative editing distance among the pairs with the top 5% of combined mapping rates.

**Stage 3.** To resolve ambiguities in the choice of haplotypes AltHapAlignR constructs a weighted directed graph of genes, incorporating the eight MHC reference haplotypes is constructed with one node for each gene and an edge connecting genes that are adjacent to each other on a given haplotype (Fig. 1 and Supplementary Fig. S1). Let M(*g*1, *g*2) be the average combined mapping rate of genes *g*1 and *g*2 in all haplotype pairs, then the weight of edge (*g*1, *g*2) is defined as:

(1) w(g1,g2)=1/−(log (1−(M(g1,g2)))).

In addition, two pseudo nodes are introduced to mark the start and end of the haplotypes. To select best haplotype pairs that minimize haplotype switching, edges with different haplotypes were penalized. The chosen penalty of 0.0028 corresponds to the standard error of the mean of combined mapping rates from the difference between true haplotypes and predicted haplotypes determined by a simulation study. Dijkstra’s algorithm, as implemented in the R package ‘igraph’ (Csardi and Nepusz, 2006), was employed to find the shortest path through this graph to derive suitable pairs of reference haplotypes (Fig. 1A).

**Output.** The main output of AltHapAlignR is a file with the combined mapping rates of all haplotype pairs, providing raw read counts of both haplotypes and genes. Gene counts from this file can be combined with data for non-MHC genes for genome-wide gene expression analyses. In addition, AltHapAlignR produces a file with three figures: a heat map providing an overview of mapping results, a table with combined mapping rates of best haplotype pairs and a bar plot with haplotype and gene counts (Fig. 1B). An example of the full output for all MHC genes is shown in Supplementary Figure S2. Optionally, a file annotating each read with its assignment to gene features and haplotypes can be produced.

### 2.2 Estimating expression of genes and haplotypes

We quantified features of transcripts and genes using GENCODE (Harrow *et al.*, 2012) Release 21 (GRCh38) annotations. Only protein coding transcripts with verified and manually annotated loci were used based on a gene type and a level feature information in GENCODE. Genomic locations of genes were restricted to filtered transcript location. Genes were removed from the analysis if they had a low proportion of uniquely mapped reads (< 80%) or if they were the target gene of incorrectly mapped reads (> 20% of reads for a given gene), as determined by a simulation study (Supplemental Table S1). These included genes known to be highly homologous such as the heat shock genes *HSPA1A* and *HSPA1B* and complement genes *C4A* and *C4B*. In total, expression was estimated for 135 genes in the 8 MHC haplotypes.

We estimated read counts for each gene by summing over the read counts of transcripts from the best pair of reference haplotypes. For comparison of haplotype expression on a per gene basis, we only used reads uniquely mapped to a single haplotype. To obtain haplotype counts for a heterozygous gene for comparison of haplotype expression across different groups, the normalized gene count was multiplied by proportion of uniquely mapped reads to each haplotype. For homozygous genes, normalized gene counts were divided by 2.

### 2.3 Data simulation

Paired-end short reads were generated based on transcript sequences from the eight MHC reference sequences. Haplotypes and transcripts were randomly selected for each gene. For each haplotype/gene pair, we generated 2000 read pairs by randomly selecting their start and end positions in transcript sequences with insert sizes between 150 and 350 bp. We produced data for five different sampling ratios (1:1, 1:1.125, 1:1.25, 1:1.5 and 1:2) between two haplotypes and for two different read lengths (50  and 100 bp).

### 2.4 Comparison with salmon

The 10 sets of simulated data were analysed with Salmon version 0.9.1 using its quasi-mapping-based mode. Read mapping was restricted to the set of protein coding genes that were used in the simulation. Simulated reads were quantified directly against the resulting index using the Salmon ‘quant’ command. Salmon’s estimate of the number of reads mapping to each transcript (NumReads) was used for transcript-level abundance. Trimport (Soneson *et al.*, 2015) was used to compute the gene-level estimates from transcript estimates. We used the read counts of genes and transcripts only matching to the simulated dataset and compared the performance of the expression estimation to ours. Identical sequences in different haplotypes were treated as the same gene. Incorrect mapping rates were computed as the expression estimates for transcripts not present on the simulated haplotypes over the total expression, aggregated at the gene level.

### 2.5 Sample preparation of synthetic heterozygote

Two of HLA-homozygous lymphoblastoid cell lines (LCLs), PGF and COX were used to produce ‘synthetic heterozygote’ samples. The COX cell line was obtained from The International Histocompatibility Working Group (IHW, ref 0922) and PGF cell line from the European Cell Culture Collection (Salisbury, UK ref 94050342); integrity and genotypes had been previously verified by DNA FISH, HLA typing, microsatellite and SNP (single nucleotide polymorphism) genotyping as described (Vandiedonck *et al.*, 2011). Cells were harvested in mid-log growth phase, total RNA extracted and quantified using the Qubit RNA assay. RNA from each of the two MHC homozygous cell lines was mixed in differing ratios before mRNA library preparation and sequencing. Libraries were generated from each of the RNA ratio mixes using polyT capture of mRNA. Once the mRNA had been selected the RNA was fragmented and then cDNA libraries were made. The cDNA was end repaired, A-tailed and adaptor-ligated before amplification for sequencing. Sequencing was performed using the Illumina GAIIx. RNA-seq data are deposited at ArrayExpress accession number E-MTAB-5870.

### 2.6 GEUVADIS RNA-seq data analysis

RNA-seq data generated as part of the GEUVADIS project (Genetic European Variation in Health and Disease, A European Medical Sequencing Consortium) was used for this study. The dataset includes RNA-seq data on LCLs established from 462 individuals from five populations: 91 CEPH (CEU), 95 Finns (FIN), 94 British (GBR), 93 Toscani (TSI) and 89 Yoruba (YRI) (Lappalainen *et al.*, 2013). Bam files for paired-end reads (2 × 75 bp) were downloaded from ArrayExpress (accession number E-GEUV-6).

We focused on analysing the expression of genes and haplotypes in the MHC region, using the strategy described earlier. To identify genes with differential expression between populations, we generated raw read counts of genes of all individuals using AltHapAlignR and these were normalized using DESeq2 (Love *et al.*, 2014). Genes with Benjamini–Hochberg adjusted *P*-value < 0.05 and fold change >2 were considered significant. We estimated expression levels of haplotypes for heterozygous genes by comparing uniquely mapped reads to two predicted haplotypes. When comparing haplotype expression within population, we only included haplotype pairs with more than 5 individuals and a read depth of at least 30 in both haplotypes.

We performed HLA-typing of all RNA-seq data using PHLAT (Bai *et al.*, 2014), which is based on SNP sites against reference sequences in IMGT/HLA database (Robinson *et al.*, 2015). Reads mapped to the best pair of haplotypes for each gene were extracted and mapped to the IMGT/HLA sequences that contain DNA sequences of HLA alleles to find a pair of the reference haplotype and the HLA type.

### 2.7 Software availability

AltHapAlignR is freely available under the terms of the LGPL-3 license at https://github.com/jknightlab/AltHapAlignR. Customized shell scripts provided in the R package for remapping can be run on a per sample basis.

## 3 Results

### 3.1 Read mapping accounting for alternate haplotypes using AltHapAlignR

We first aimed to develop an approach for RNA-seq read mapping that can take account of alternate haplotypes, using the MHC as an exemplar region. To do this, we developed a novel analytical pipeline (denoted AltHapAlignR) (Fig. 1;Supplementary Fig. S1) in which reads are aligned to the reference sequence for this region. This currently corresponds to the PGF haplotype as part of the GRCh38 reference sequence (Schneider *et al.*, 2017). Unmapped and reads mapped to chromosome 6 (chr6: 29, 580, 000-33 100 000) are then extracted and realigned to the other seven MHC reference haplotypes independently. We ensured uniqueness in mapping for a given read or pair of reads, with weighting based on editing distance to calculate mapping rates and infer the best pair of reference haplotypes.

### 3.2 Comparison of accuracy of quantification using simulated data

**Fig. 2.**
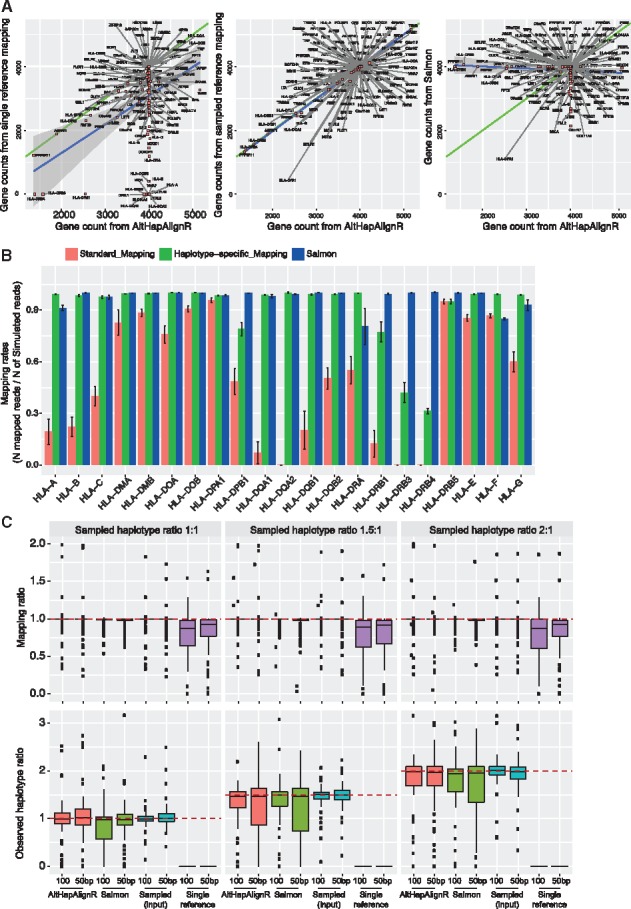
Accuracy of estimated gene and haplotype level expression using simulated data for MHC genes. (**A**) Gene counts for input simulated reads (100 bp) comparing mapped read counts using single reference, AltHapAlignR or Salmon. (**B**) Mapping rates between single reference-based mapping, AltHapAlignR or Salmon for HLA genes (error bars SEM). (**C**) Estimated mapping ratios relative to true actual sample ratios. Expected mapping ratio is indicated by a dashed line. At haplotype level, boxplots show estimated haplotype ratios with actual ratios indicated by dashed lines

We proceeded to compare our AltHapAlignR approach accounting for alternate haplotypes with a standard mapping procedure using the GRCh38 reference sequence as well as to gene expression estimates produced by Salmon (Patro *et al.*, 2017). To do this, we randomly generated simulated reads (50 and 100 bp) from up to two of the eight MHC reference haplotypes for each gene, with relative gene expression levels set at five different ratios (1:1, 1:1.125, 1:1.25, 1:1.5 and 1: 2) between any pair of haplotypes. We obtained gene counts mapped to the GRCh38 reference sequence and compared them to gene counts obtained by our AltHapAlignR method. For the latter, we estimated gene counts by summing the read count from the best pair of haplotypes. We found that the correlation between simulated and predicted gene counts using AltHapAlignR was very high (*r*^2^ 0.99), whereas the correlation obtained when using Salmon or a single reference was substantially lower (*r*^2^ 0.65 and 0.46, respectively) (Fig. 2A), with longer reads improving accuracy (Supplementary Fig. S3).

Using conventional mapping with a single reference haplotype, we found that expression levels of 85 out of 135 genes (63%) in the MHC were estimated to have an expression level at least 10% below the nominal value. Salmon performed better with 31 genes (23%) producing low expression estimates. AltHapAlignR compared favorably to this with only 13 genes (10%) producing low estimates. This includes six classical HLA genes and seven other HLA genes, with some of these failing to attract any reads (Fig. 2B, Supplementary Figs. S3B and S4A). At the haplotype level, we found that there was a high level of correlation between estimated and simulated median expression ratios using AltHapAlignR (*r*^2^ 0.95) for best matching haplotypes (Fig. 2C and Supplementary Fig. S4B). We also found that using longer reads improved the accuracy of estimated expression ratios (Supplementary Fig. S4B).

### 3.3 Generation and quantification of synthetic samples comprising different allelic ratios

**Fig. 3.**
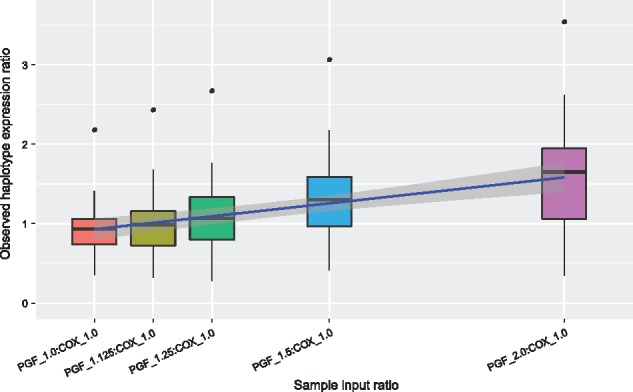
Haplotype ratios of gene expression for synthetic heterozygote samples. Box plots present estimated haplotype ratios of genes from synthetic heterozygote samples prepared in five different haplotype ratios (1:1, 1:1.125, 1:1.25, 1:1.5 and 1:2)

We validated our approach experimentally by generating ‘synthetic heterozygote’ samples in which we combined total RNA from LCLs homozygous for two MHC haplotypes (PGF and COX) in five different allelic ratios (1:1, 1:1.125, 1:1.25, 1:1.5 and 1:2) and used these to generate RNA-seq sequencing libraries. We calculated expected haplotype ratios for heterozygous genes, which had uniquely mapped reads with minimum read depth of 30 in both haplotypes. We were able to quantify haplotype-specific differences (Fig. 3) with a correlation observed between input and observed median ratios (0.997). Median ratios were consistently lower than expected, however, scaling by the ratio observed in the 1:1 sample reduced the observed deviation from expected (Supplementary Fig. S5). Observed differences may reflect biological differences between the gene expression for the two haplotypes or the difficulty of accurate quantification of RNA abundance at the time of mixing RNA from the two cell lines.

### 3.4 Application of AltHapAlignR to large-scale RNA-seq data from diverse populations

**Fig. 4.**
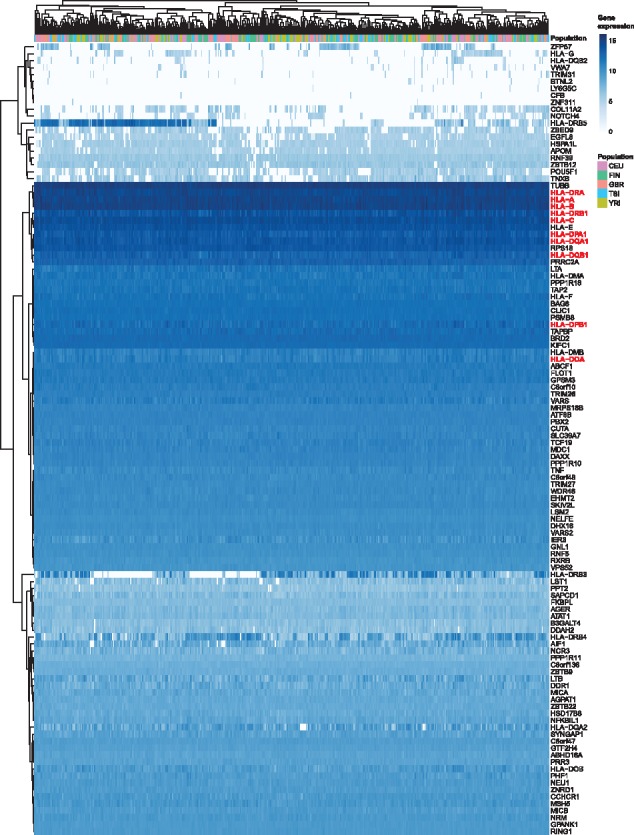
Heat map diagram of MHC gene expression analysed by AltHapAlignR for GEUVADIS cohort. Gene level expression for 114 genes in the MHC from 462 individuals in five populations shown. Each column represents an individual, each row a single gene (classical HLA genes highlighted). Normalized log2 expression levels shown (white, low intensities; dark shading, high intensities)

We next aimed to investigate differential expression of genes and alleles for MHC haplotypes encountered in diverse populations. We applied AltHapAlignR to the GEUVADIS dataset comprising deep RNA-seq data for 462 LCLs established from individuals from five different populations (Lappalainen, *et al.*, 2013). Gene expression was variable with high levels of expression seen, notably in classical HLA genes (Fig. 4) (Supplemental Table S2) with six genes (*HLA-A*, *HLA-B*, *HLA-C*, *HLA-DQA1*, *HLA-DQB1* and *HLA-DRB1*) accounting for 25–47% of expression in all genes in the MHC across all individuals. Genes showing significant variation in expression between individuals included genes only found on specific haplotypes (such as *HLA-DRB5*) and others with regulatory genetic variants modulating transcription [such as *ZFP57* (Plant *et al.*, 2014)] or mRNA stability [such as *HLA-G* (Rousseau *et al.*, 2003)].

**Fig. 5.**
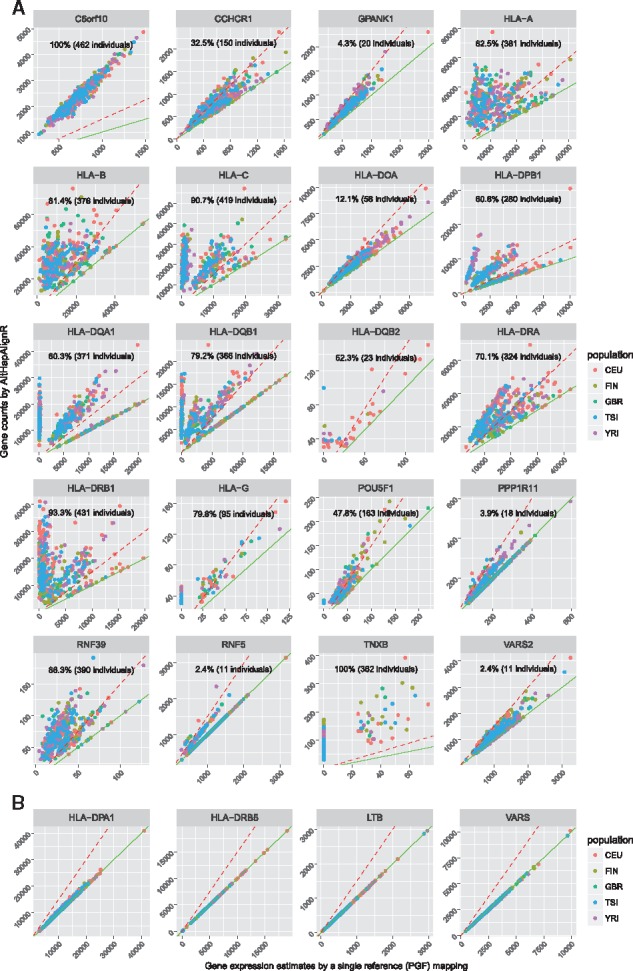
Variation in estimated gene expression estimates for GEUVADIS cohort by mapping method. Read counts by single reference-based mapping (x-axis) and by AltHapAlignR (y-axis) shown, each dot indicating an individual. (**A**) Scatter plots for 20 genes showing 1.5-fold change (red dashed line) with lower expression in single reference mapping versus AltHapAlignR. (**B**) Scatter plots illustrating genes with similar estimated expression

We compared gene level expression estimated by single reference-based mapping with that achieved using AltHapAlignR (Fig. 5, Supplementary Fig. S6). We found significant differences in expression with 20 genes showing 1.5-fold lower level of gene expression in the single reference-based mapping at least in ten individuals (Fig. 5A, Supplemental Table S3). These included the majority of classical class I (*HLA-A*, *HLA-B*, *HLA-C* and *HLA-G*) and class II (*HLA-DOA*, *HLA-DPB1*, *HLA-DQA1*, *HLA-DQB1*, *HLA-DQB2*, *HLA-DRA* and *HLA-DRB1*) genes.

In contrast, *HLA-DPA1* showed consistent gene expression from the two mapping procedures (Fig. 5B). For this gene, all the reference haplotypes carry the same allele (DPA1*01:03:01), whereas the HLA genes that showed significant differences in expression estimates have different alleles across different haplotypes (Robinson *et al.*, 2015).

**Fig. 6.**
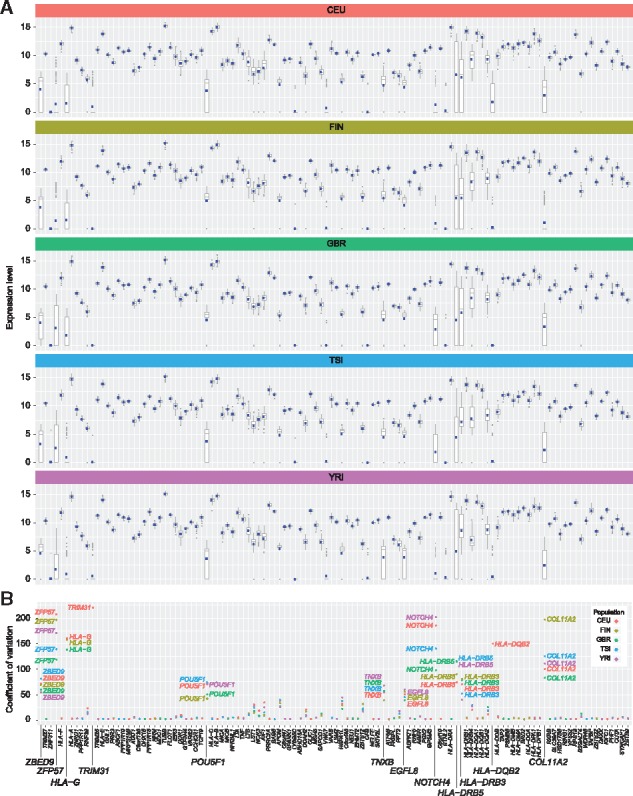
Variation in gene level expression across and within populations. (**A**) Box plots of normalized log2 read counts for each gene across five populations from the GEUVADIS cohort. (**B**) Coefficient of variation for each gene by population

We explored population differences in gene level expression using the quantification achieved through AltHapAlignR. We found that comparing median values of gene expression in each population, the correlation coefficient was >0.94 between all population pairs (Supplementary Fig. S7). While there was a high correlation in overall gene expression between different populations, there were notable outliers that showed over 4-fold difference between populations, involving *HLA-DRB3*, *HLA-DRB4*, *HLA-DRB5*, *NOTCH4* and *COL11A2* (Supplementary Fig. S7 and Table S4). These five genes are also among those showing the broadest range of expression within populations (Fig. 6). Other genes showing variation in expression within populations included HLA genes *HLA-DQA2*, *HLA-DQB2* and *HLA-G* and non-HLA genes *EGFL8*, *POU5F1*, *TNXB*, *ZBED9* and *ZFP57* (Fig. 6).

**Fig. 7.**
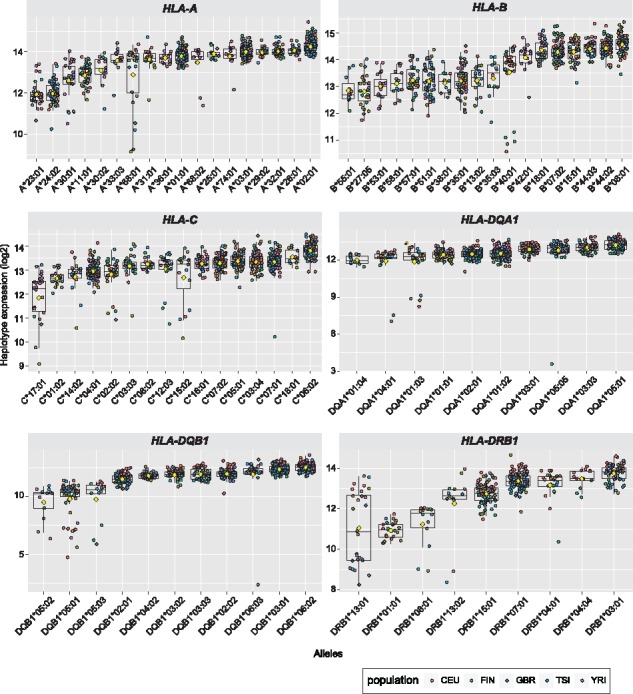
Gradients of expression across allelic lineages of six classical HLA genes (HLA-A, HLA-B, HLA-C, HLA-DQA1, HLA-DQB1 and HLA-DRB1) in the GEUVADIS cohort. Expression values plotted by allele (two for each individual) and coloured by population. Allelic lineages with >=10 individuals are shown. Yellow diamonds represent average expression levels for each allelic lineage of their role in disease, which may involve effects not only at a structural level but also differences in levels of gene expression

Classical HLA genes have a large number of different alleles and the extent to which these vary in terms of gene expression is an unresolved question although evidence suggests significant variation (Apps *et al.*, 2015) with potentially important consequences for function and disease risk (Apps *et al.*, 2013). We inferred high resolution HLA types from RNA-seq data using PHLAT (Bai *et al.*, 2014) in which reads mapping to the best pair of haplotypes for each gene were extracted and mapped to IMGT/HLA allelic sequences to find a pair of the reference haplotype and the HLA type using a likelihood based ranking. We observed gradients in expression for the alleles of the different classical HLA genes (Fig. 7).

## 4 Discussion

It is well-established that read mapping algorithms are biased towards reference alleles (Degner *et al.*, 2009). For genes that harbor multiple non-reference alleles, as is the case for many MHC genes, this can lead to a significant under-estimation of gene expression. The approach outlined here alleviates this by allowing for the inclusion of multiple reference sequences. AltHapAlignR enables finding the best match or closest haplotypes for all genes annotated in the current references and estimating their expression using RNA-seq data. We demonstrated the utility of our approach using eight MHC reference haplotypes. The ability to comprehensively analyse expression of genes in the MHC will inform understanding of their role in disease, which may involve effects not only at a structural level but also differences in levels of gene expression.

We found evidence that some MHC genes show strong evidence of allele specific expression with the same allele (as identified by the best matching reference haplotype) exhibiting consistent expression patterns across populations. This effect is more pronounced than any population specific differences we observed in the GEUVADIS data. Indeed, it seems likely that differential expression observed between populations is largely due to differences in allele frequencies. For example, the observed variation in gene expression within and between populations involving functional paralogous *HLA-DRB* genes is likely to be accounted for by the variable number of HLA-DRB loci dependent on haplotype that vary in frequency across populations (Gonzalez-Galarza *et al.*, 2015; Zhang *et al.*, 2017). This variation is reflected in the occurrence of these genes among the reference haplotypes (*HLA-DRB1* is present in seven out of the eight available reference haplotypes, whereas *HLA-DRB3* is only present on COX and QBL, *HLA-DRB4* on MCF and SSTO and *HLA-DRB5* on PGF) and in population studies where, for example, African ancestry populations have high frequency of *HLA-DRB3* and low *HLA-DRB4* (Zhang *et al.*, 2017) consistent with our observations at the level of gene expression. We also provide estimates of differences in gene expression specific to individual HLA types involving six classical HLA genes, with HLA typing information extracted from RNA-seq data by HLA typing tools. We find that while expression of the closest matched haplotypes to individual HLA types is broadly comparable across allelic lineages there are some differences in expression.

Our AltHapAlignR method is directly applicable to many publicly available RNA sequencing datasets for which expression of genes in the MHC region has not been studied. It is, however, not limited to the analysis of the MHC. Alternative haplotypes for many regions of the human genome are now available (Church *et al.*, 2015) and could be integrated into an RNA-seq analysis using AltHapAlignR, enabling more accurate estimation of gene expression and opportunities for analysis of allele-specific expression. In addition, AltHapAlignR provides bam files filtered with reads assigned to predicted haplotypes, which allows detecting mutations. This can improve accuracy of somatic mutation detection to understand the implicated dysfunction in immune evasion as somatic mutations of genes in the MHC region were observed to be a frequent process in some tumor types (Lawrence *et al.*, 2014). For any of these regions additional haplotype sequences and improved annotation can be easily integrated by users into the AltHapAlignR R package.

AltHapAlignR was designed to take advantage of existing reference haplotypes and uses the available information to produce less biased estimates of gene expression for highly polymorphic genes. It is not intended to derive novel haplotypes or identify specific alleles of a given gene. Other technologies, especially those utilizing long sequencing reads, are better suited to this task. However, we note that although such technologies will improve haplotype prediction and facilitate the assembly of sample haplotypes this is still costly and their utility for RNA-seq analyses remains limited for now. Moreover, our method will allow mining of the large number of existing RNA-seq datasets for more accurate estimation of gene expression in highly polymorphic loci that are typically of significant research interest due to disease association and is applicable to other genomic regions as alternate reference haplotypes become available.

## Supplementary Material

Supplementary Data
