# Supplementary material for: AltHapAlignR: improved accuracy of RNA-seq analyses through the use of alternative haplotypes
Source: Bioinformatics. 2018 Mar 5;34(14):2401–8. doi: 10.1093/bioinformatics/bty125 (PMC6041798; doi:10.1093/bioinformatics/bty125)
Supplement: Supplementary Data [file bty125_supp.zip › bty125-suppl_data/lee_supplemental_revision.pdf]

Supplemental Materials for

Improved accuracy for RNA-seq analyses through the use of alternative haplotypes

Wanseon Lee, Katharine Plant, Peter Humburg, Julian C Knight

Table of contents

Supplemental Figures S1-S7

Supplemental Tables S1-S4

For a gene G1, calculating rates of gene counts

Reads	H1 + H2	H1 + H3	H2 + H3	H1 + H1	H2 + H2	H3 + H3
1	0	1	1	0	0	1
2	0	0	1	0	0	1
3	1	1	0	1	0	0
4	0	1	1	1	1	1
5	0	1	1	1	1	0
6	1	0	1	0	1	1
…						
100	1	1	1	1	0	1
Mapping rates	57/100	78/100	96/100	20/100	45/100	70/100

For a gene G1, calculating mapping rates

	Editing distance (number of mismatch)					
Reads	H1 + H2	H1 + H3	H2 + H3	H1 + H1	H2 + H2	H3 + H3
1	-	0	0	-	-	0
2	-	-	1	-	-	1
3	2	2	-	2	-	-
4	-	1	0	1	0	0
5	-	1	1	3	1	-
6	1	-	1	-	1	1
…						
100	1	1	1	2	-	0
Mapping rates	0.91	0.96	0.98	0.95	0.97	0.99

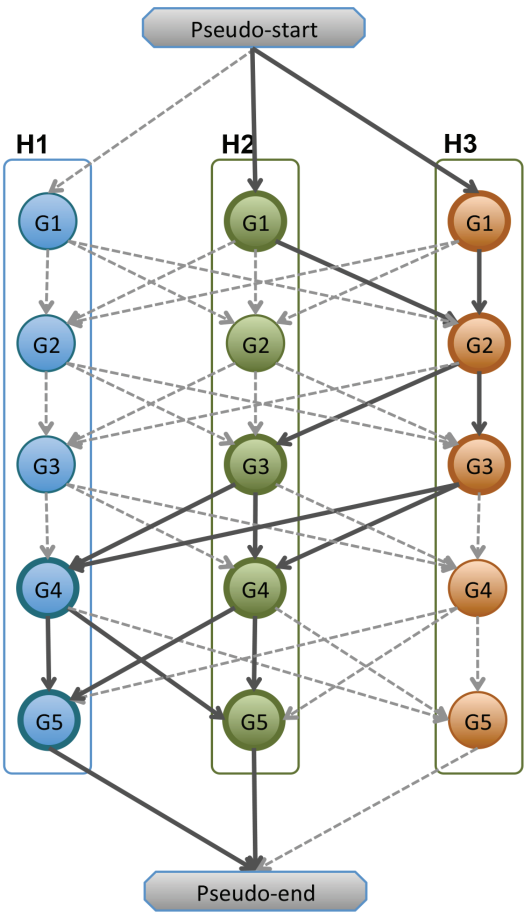

Supplemental Figure S1. An example of calculating mapping rates and a directed graph structure for the shortest path study. (**A**) For each gene, gene counts and mapping rates based on an editing distance are calculated. (**B**) Each node represents a gene and edges connect adjacent genes across all haplotypes. Two pseudo nodes were added to mark the start and end of the graph. The best pair of haplotypes for each gene is selected based on mapping rates. The weight of edge was calculated by using the averaged combined mapping rate of two adjacent genes to connect them each other as described in the Methods. The solid lines are selected edges in the constructed edges (dashed lines) in the graph.

Supplemental Figure S2. AltHapAlignR output using synthetic heterozygote data (PGF and COX 1:1 ratio) for all MHC region genes. Haplotype prediction and mapping rates (left panel). Illustrated for each MHC gene (y-axis) with respect to each of eight known haplotypes and presented as a heat map. Numbers in each cell are mapping rates. Predicted haplotypes are highlighted with a red border. Empty cells represent genes not annotated in the given haplotype. Combined mapping rates from the predicted haplotypes (middle panel). Each mapping rate in the first column is the read counts of the gene in the predicted haplotype(s) divided by the total read count of the gene across all haplotypes. Mismatching mapping rates of predicted haplotypes are in the second column. Pink and grey colors are genes predicted as heterozygous and homozygous respectively. Gene counts (right panel). Bar plots show the raw read counts for each gene.

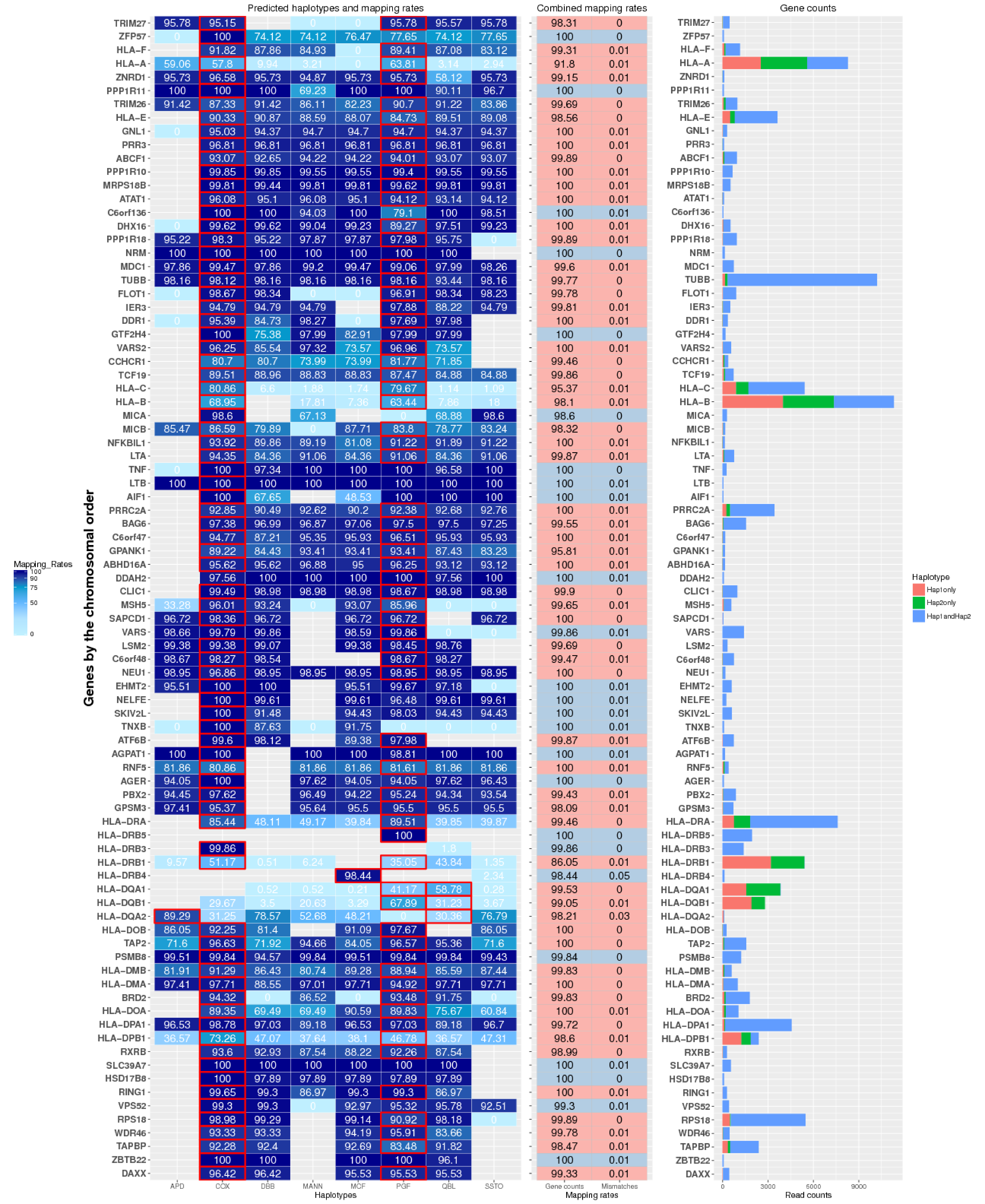

Predicted haplotypes and mapping rates
Combined mapping rates
Gene counts
Genes by the chromosomal order
Mapping_Rates
100
90
75
50
0
TRIM27
ZFP57
HLA-F
HLA-A
ZNRD1
PPP1R11
TRIM26
HLA-E
GNL1
PRR3
ABCF1
PPP1R10
MRPS18B
ATAT1
C6orf136
DHX16
PPP1R18
NRM
MDC1
TUBB
FLOT1
IER3
DDR1
GTF2H4
VARS2
CCHCR1
TCF19
HLA-C
HLA-B
MICA
MICB
NFKBIL1
LTA
TNF
LTB
AIF1
PRRC2A
BAG6
C6orf47
GPANK1
ABHD16A
DDAH2
CLIC1
MSH5
SAPCD1
VARS
LSM2
C6orf48
NEU1
EHMT2
NELFE
SKIV2L
TNXB
ATF6B
AGPAT1
RNF5
AGER
PBX2
GPSM3
HLA-DRA
HLA-DRB5
HLA-DRB3
HLA-DRB1
HLA-DRB4
HLA-DQA1
HLA-DQB1
HLA-DQA2
HLA-DOB
TAP2
PSMB8
HLA-DMB
HLA-DMA
BRD2
HLA-DOA
HLA-DPA1
HLA-DPB1
RXRB
SLC39A7
HSD17B8
RING1
VPS52
RPS18
WDR46
TAPBP
ZBTB22
DAXX
APD
COX
DBB
MANN
MCF
PGF
QBL
SSTO
Haplotypes
Gene counts
Mismatches
Mapping rates
0
3000
6000
9000
Read counts
Haplotype
Hap1only
Hap2only
Hap1andHap2

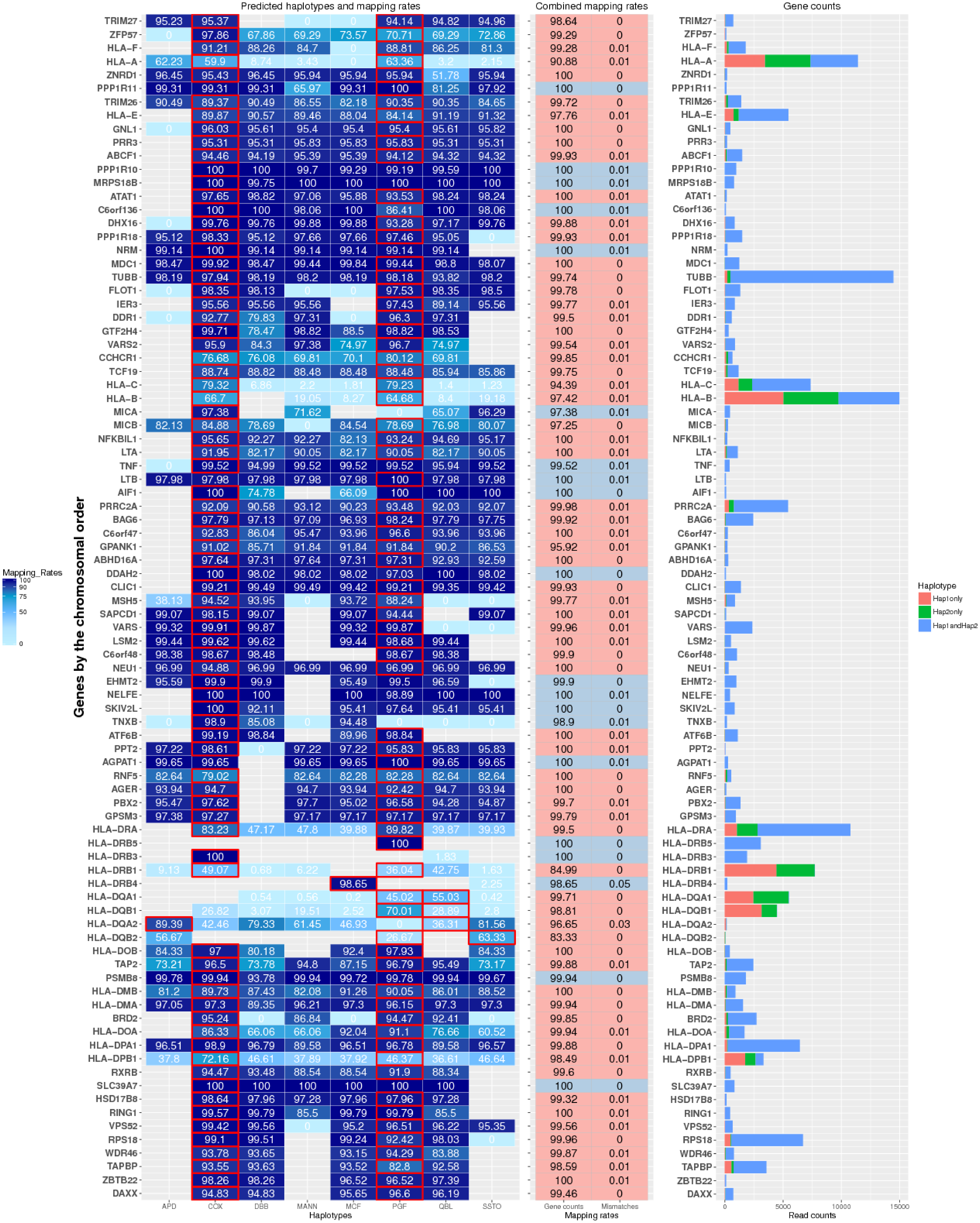
Predicted haplotypes and mapping rates
Combined mapping rates
Gene counts
Genes by the chromosomal order
Mapping_Rates
100
90
75
50
0
APD
COX
DBB
MANN
MCF
PGF
QBL
SSTO
Haplotypes
Gene counts
Mismatches
Mapping rates
0
5000
10000
15000
Read counts
Haplotype
Hap1only
Hap2only
Hap1andHap2
TRIM27
ZFP57
HLA-F
HLA-A
ZNRD1
PPP1R11
TRIM26
HLA-E
GNL1
PRR3
ABCF1
PPP1R10
MRPS18B
ATAT1
C6orf136
DHX16
PPP1R18
NRM
MDC1
TUBB
FLOT1
IER3
DDR1
GTF2H4
VARS2
CCHCR1
TCF19
HLA-C
HLA-B
MICA
MICB
NFKBIL1
LTA
TNF
LTB
AIF1
PRRC2A
BAG6
C6orf47
GPANK1
ABHD16A
DDAH2
CLIC1
MSH5
SAPCD1
VARS
LSM2
C6orf48
NEU1
EHMT2
NELFE
SKIV2L
TNXB
ATF6B
PPT2
AGPAT1
RNF5
AGER
PBX2
GPSM3
HLA-DRA
HLA-DRB5
HLA-DRB3
HLA-DRB1
HLA-DRB4
HLA-DQA1
HLA-DQB1
HLA-DQA2
HLA-DQB2
HLA-DOB
TAP2
PSMB8
HLA-DMB
HLA-DMA
BRD2
HLA-DOA
HLA-DPA1
HLA-DPB1
RXRB
SLC39A7
HSD17B8
RING1
VPS52
RPS18
WDR46
TAPBP
ZBTB22
DAXX

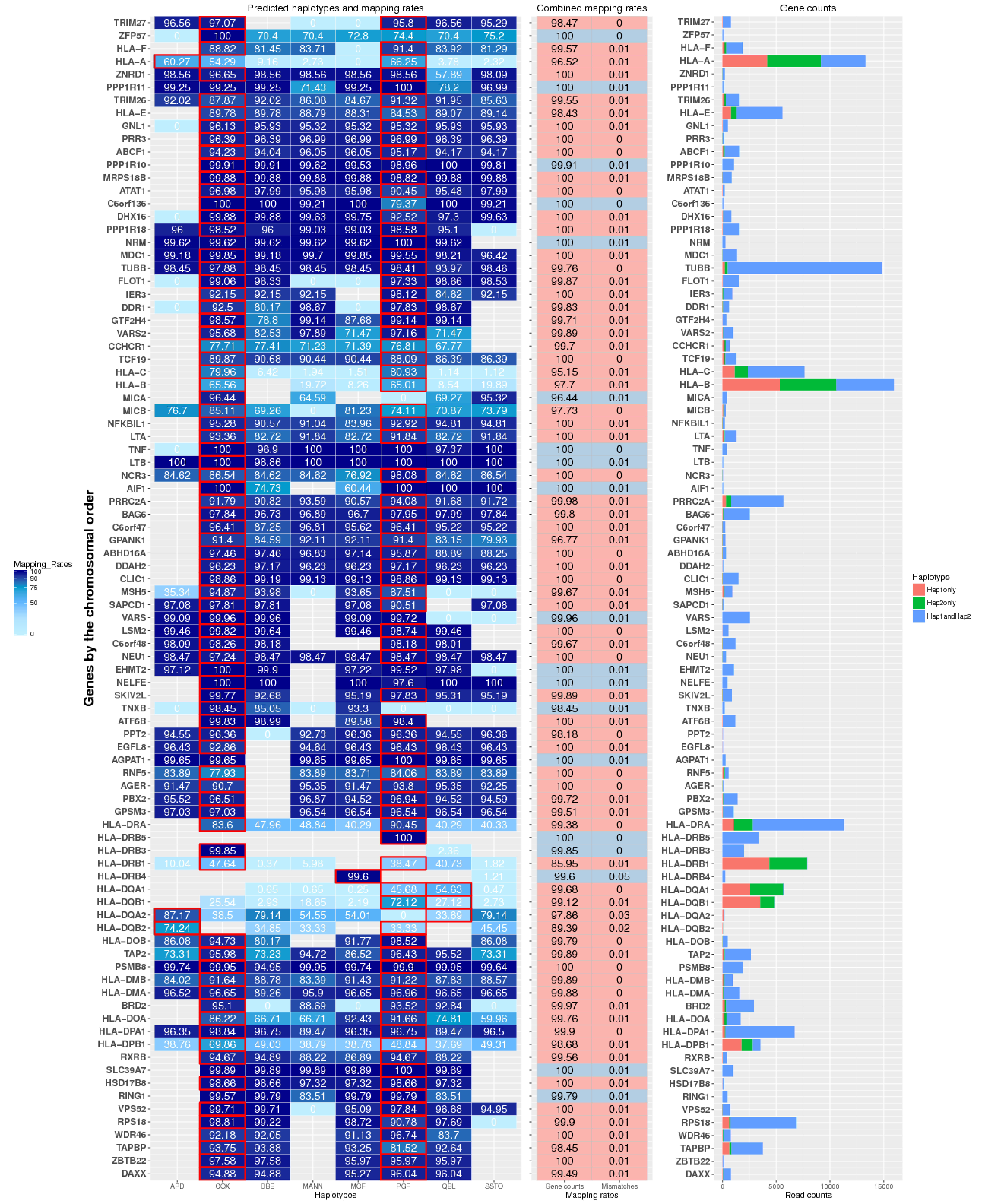
Predicted haplotypes and mapping rates
Combined mapping rates
Gene counts
Genes by the chromosomal order
TRIM27
ZFP57
HLA-F
HLA-A
ZNRD1
PPP1R11
TRIM26
HLA-E
GNL1
PRR3
ABCF1
PPP1R10
MRPS18B
ATAT1
C6orf136
DHX16
PPP1R18
NRM
MDC1
TUBB
FLOT1
IER3
DDR1
GTF2H4
VARS2
CCHCR1
TCF19
HLA-C
HLA-B
MICA
MICB
NFKBIL1
LTA
TNF
LTB
NCR3
AIF1
PRRC2A
BAG6
C6orf47
GPANK1
ABHD16A
DDAH2
CLIC1
MSH5
SAPCD1
VARS
LSM2
C6orf48
NEU1
EHMT2
NELFE
SKIV2L
TNXB
ATF6B
PPT2
EGFL8
AGPAT1
RNF5
AGER
PBX2
GPSM3
HLA-DRA
HLA-DRB5
HLA-DRB3
HLA-DRB1
HLA-DRB4
HLA-DQA1
HLA-DQB1
HLA-DQA2
HLA-DQB2
HLA-DOB
TAP2
PSMB8
HLA-DMB
HLA-DMA
BRD2
HLA-DOA
HLA-DPA1
HLA-DPB1
RXRB
SLC39A7
HSD17B8
RING1
VPS52
RPS18
WDR46
TAPBP
ZBTB22
DAXX
APD
COX
DBB
MANN
MCF
PGF
QBL
SSTO
Haplotypes
Gene counts
Mismatches
Mapping rates
0
5000
10000
15000
Read counts
Mapping_Rates
100
90
75
50
0
Haplotype
Hap1only
Hap2only
Hap1andHap2

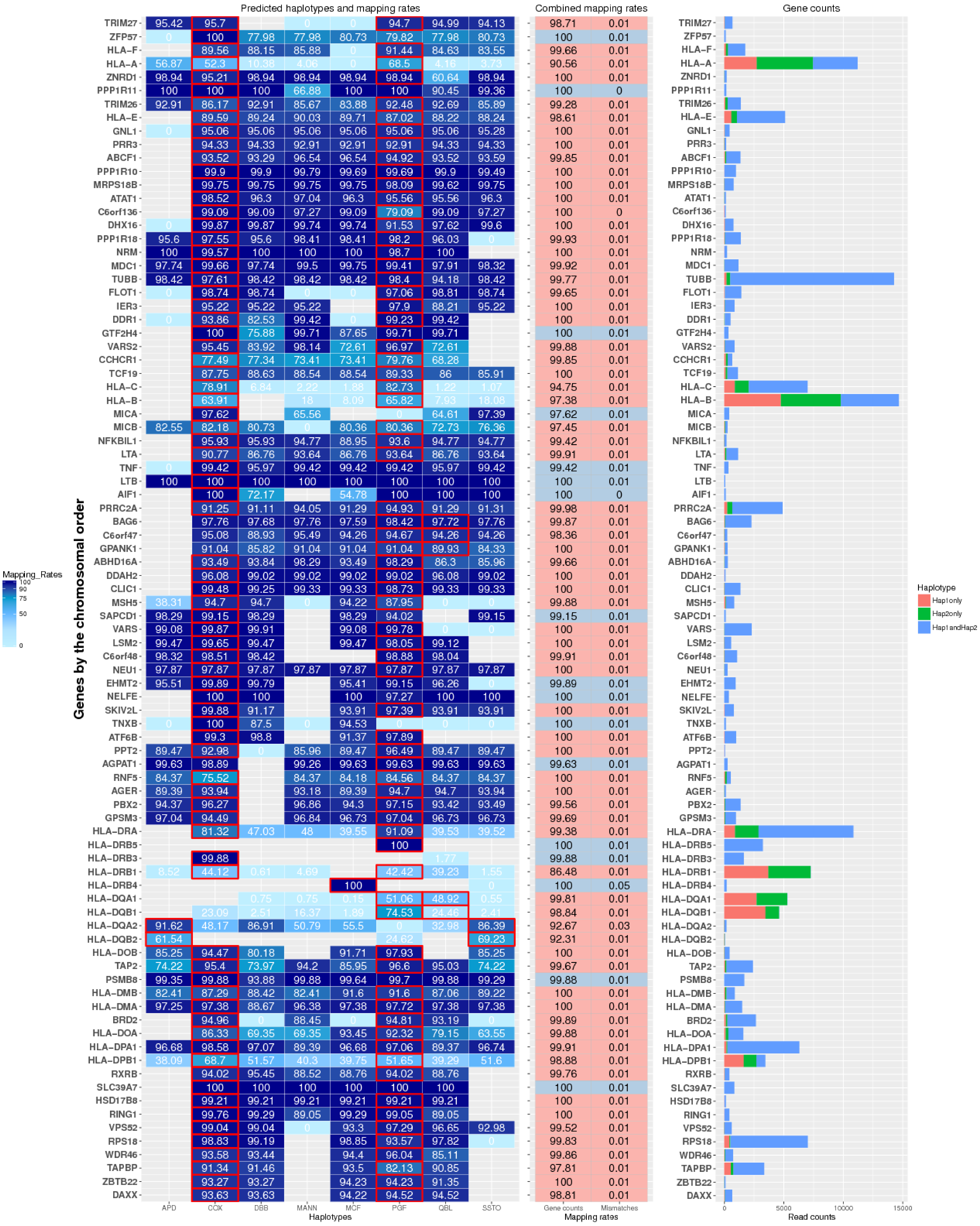

Predicted haplotypes and mapping rates
Combined mapping rates
Gene counts
Genes by the chromosomal order
Haplotypes
APD
COX
DBB
MANN
MCF
PGF
QBL
SSTO
Mapping rates
Gene counts
Mismatches
Read counts
Mapping_Rates
Haplotype
Hap1only
Hap2only
Hap1andHap2

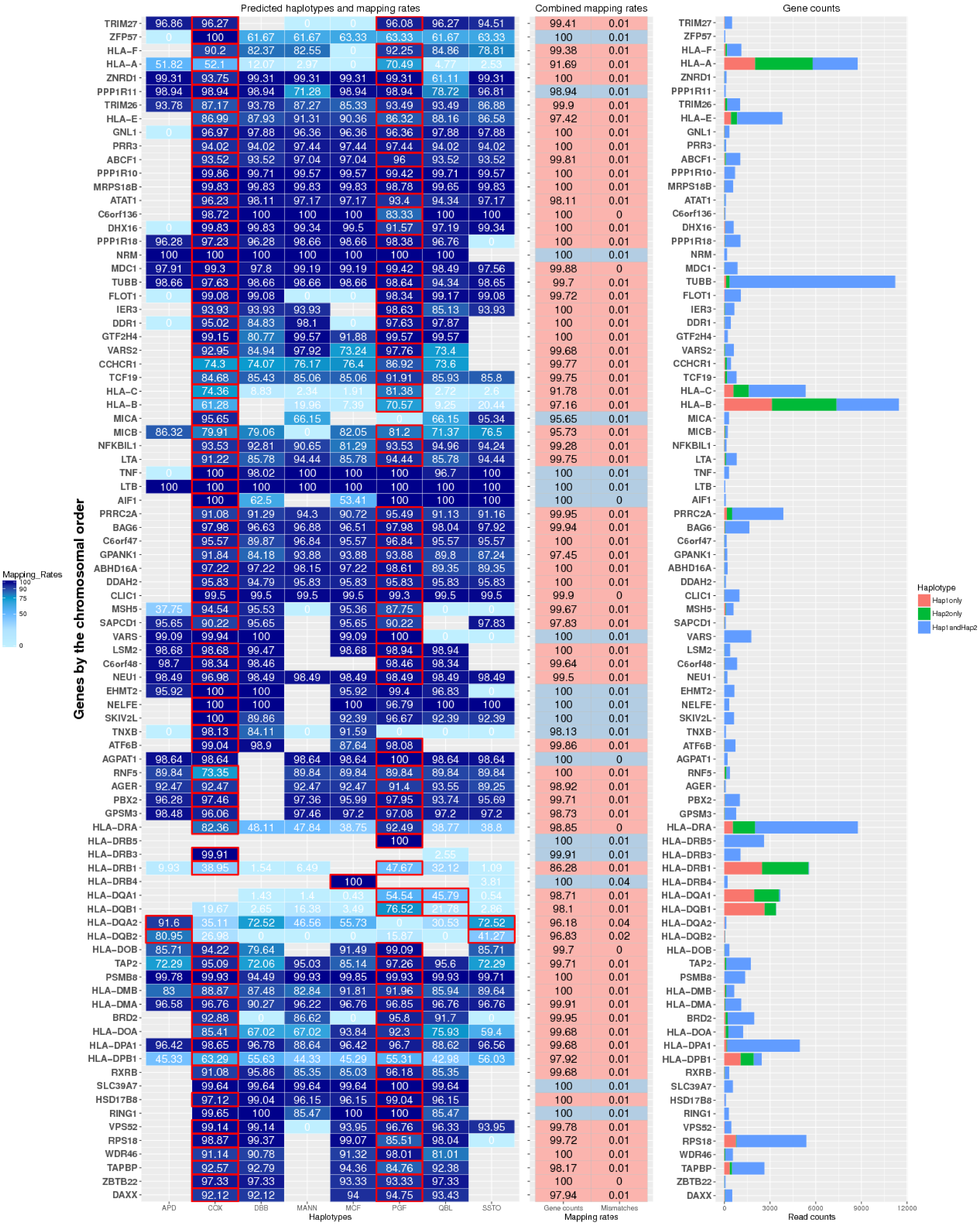
Predicted haplotypes and mapping rates
Combined mapping rates
Gene counts
Genes by the chromosomal order
Mapping_Rates
100
90
75
50
0
Haplotype
Hap1only
Hap2only
Hap1andHap2
APD
COX
DBB
MANN
MCF
PGF
QBL
SSTO
Haplotypes
Gene counts
Mismatches
Mapping rates
0
3000
6000
9000
12000
Read counts
TRIM27
ZFP57
HLA-F
HLA-A
ZNRD1
PPP1R11
TRIM26
HLA-E
GNL1
PRR3
ABCF1
PPP1R10
MRPS18B
ATAT1
C6orf136
DHX16
PPP1R18
NRM
MDC1
TUBB
FLOT1
IER3
DDR1
GTF2H4
VARS2
CCHCR1
TCF19
HLA-C
HLA-B
MICA
MICB
NFKBIL1
LTA
TNF
LTB
AIF1
PRRC2A
BAG6
C6orf47
GPANK1
ABHD16A
DDAH2
CLIC1
MSH5
SAPCD1
VARS
LSM2
C6orf48
NEU1
EHMT2
NELFE
SKIV2L
TNXB
ATF6B
AGPAT1
RNF5
AGER
PBX2
GPSM3
HLA-DRA
HLA-DRB5
HLA-DRB3
HLA-DRB1
HLA-DRB4
HLA-DQA1
HLA-DQB1
HLA-DQA2
HLA-DQB2
HLA-DOB
TAP2
PSMB8
HLA-DMB
HLA-DMA
BRD2
HLA-DOA
HLA-DPA1
HLA-DPB1
RXRB
SLC39A7
HSD17B8
RING1
VPS52
RPS18
WDR46
TAPBP
ZBTB22
DAXX

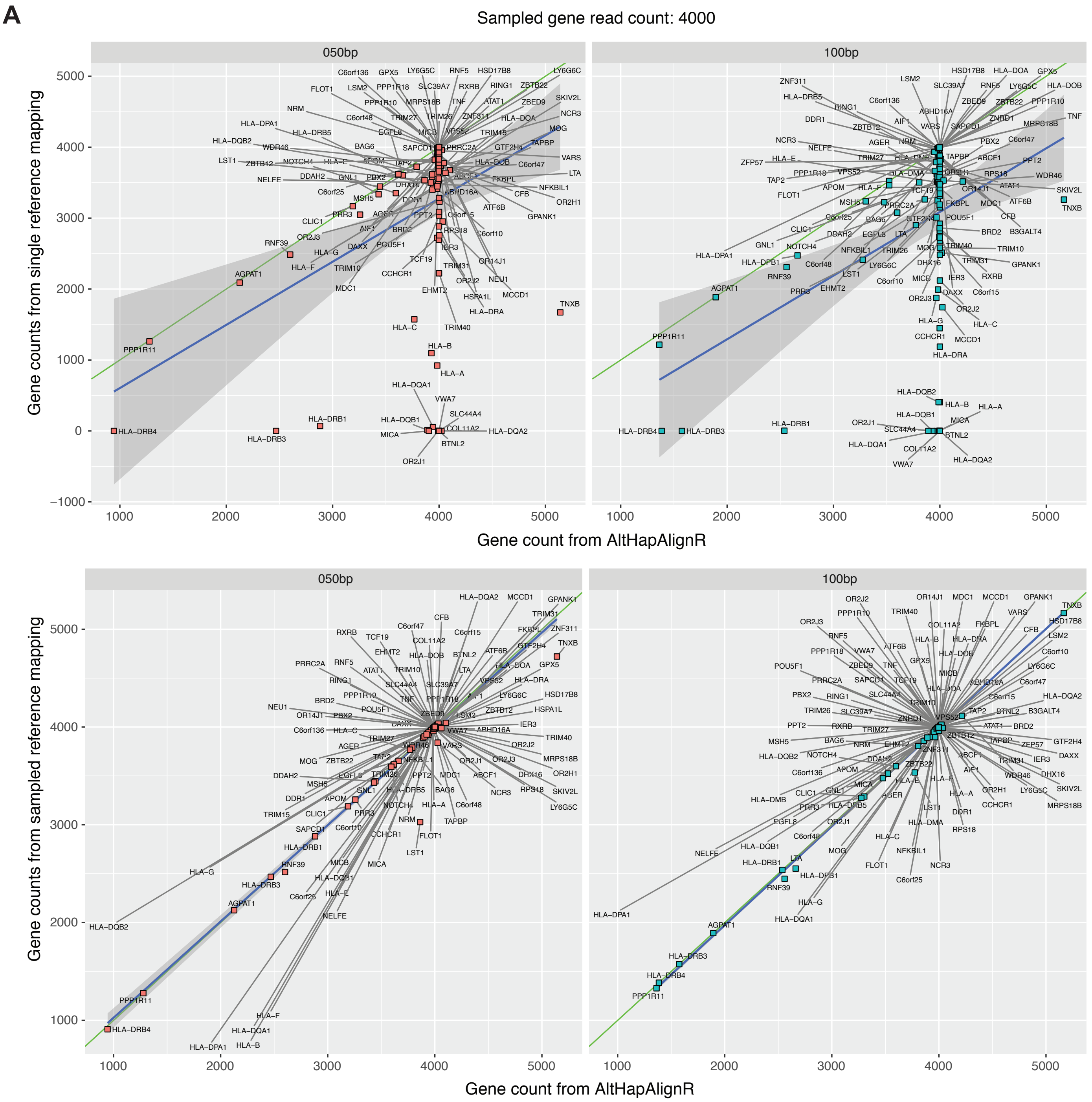

Supplemental Figure S3. Further data on accuracy of estimation of expression at gene and haplotype level using simulated data for MHC (**A**) Gene count comparison by different mappings. The correlation between simulated and predicted estimates was 0.99 (two graphs in the second row), whereas the correlation between a single reference estimates and predicted estimates was 0.46 (two graphs in the first row). (**B**) Mapping ratios of genes between AltHapAlignR and the single reference-based mapping. Mapping ratios are log2 values of read counts divided by sampled read counts for each gene. Genes with underestimated gene expression are shown in a red box.

B

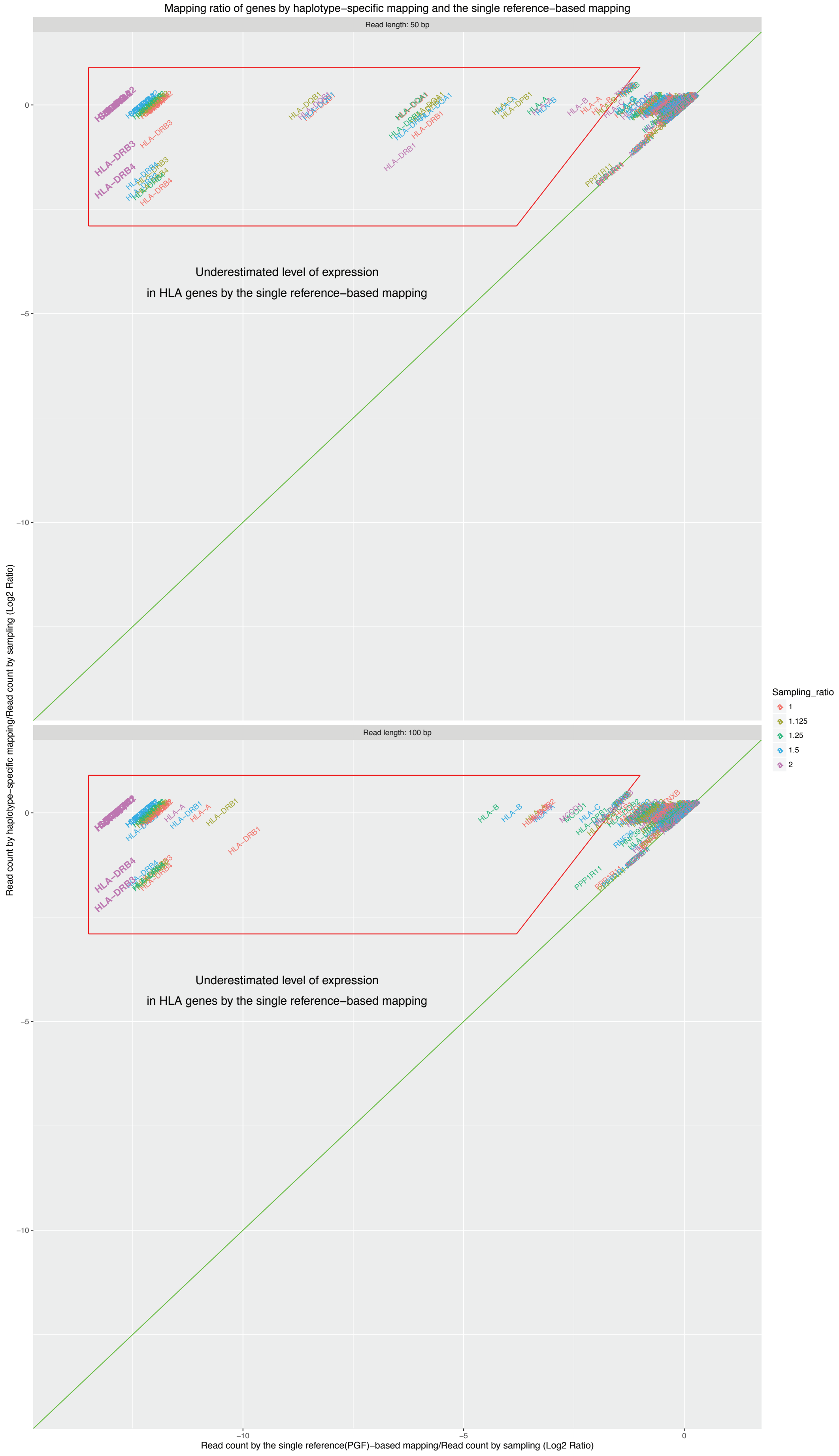

Supplemental Figure S3B

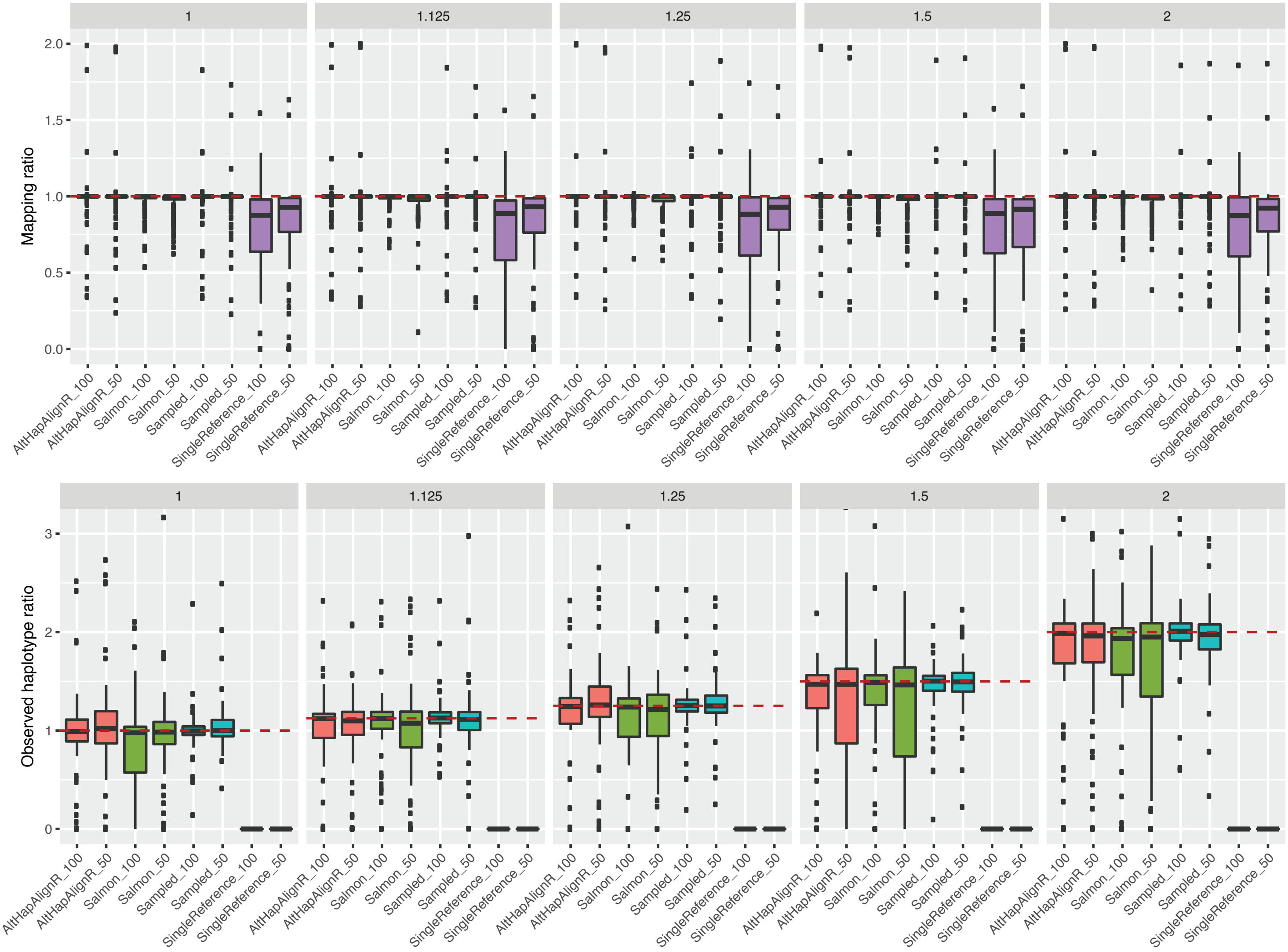

Supplemental Figure S4. Mapping ratios of genes and haplotypes in the simulated data. (A) For five different haplotype ratios with two different read length in the simulated data, box plots show mapping ratio of genes by AltHapAlignR, by Salmon, by sampled input haplotypes and by single reference-based mapping (the PGF haplotype). Perfect mapping ratio is 1 (red dash line). **(B)** Haplotype ratios of the simulated data. Red dashed lines are ratios of haplotypes in the simulated data.

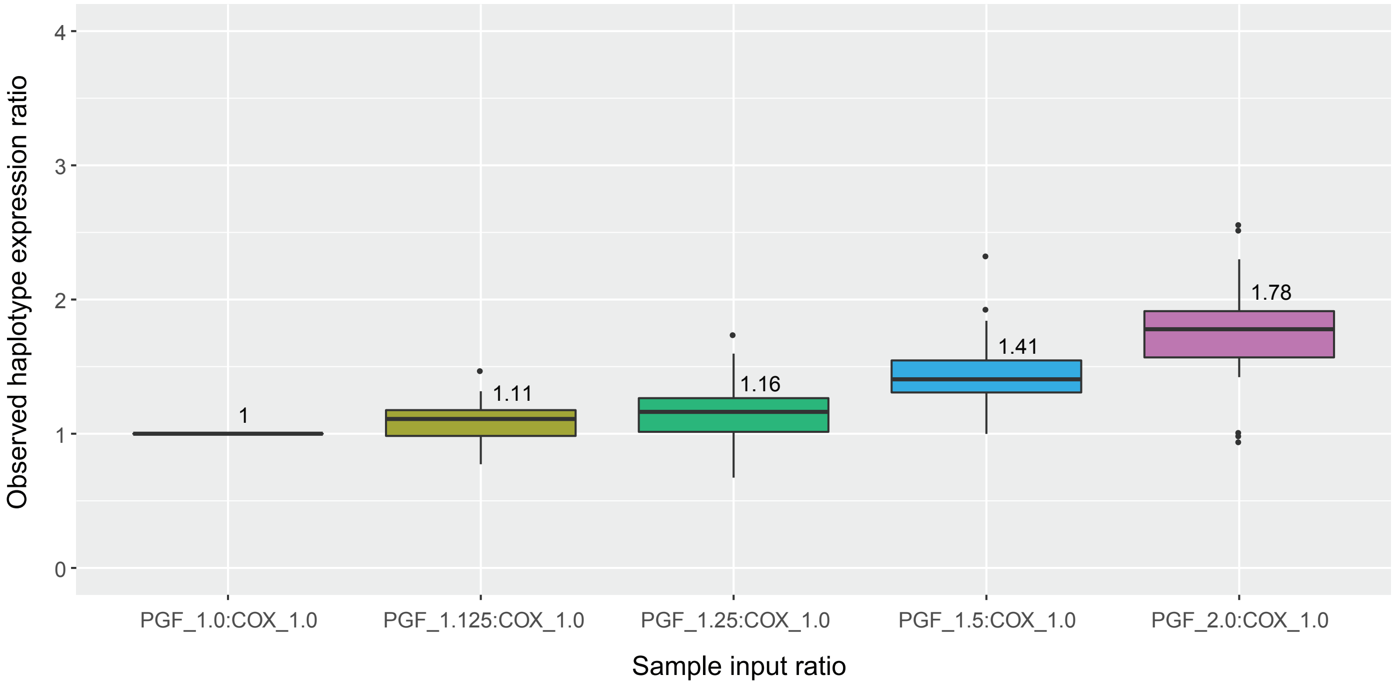

Supplemental Figure S5. Haplotype ratios of gene expression for synthetic heterozygote samples. Box plots present estimated haplotype ratios of genes (y-axis) from synthetic heterozygote samples prepared with five different haplotype ratios (1:1, 1:1.125, 1:1.25, 1:1.5 and 1:2) and scaled by 1:1 sample (x-axis).

Supplemental Figure S6. Scatterplots of gene expression estimates comparing AltHapAlignR with a single reference-based mapping for all MHC genes analysed. Raw read counts obtained by the single reference-based mapping and AltHapAlignR are shown on the X and Y-axis respectively. Each dot indicates different individual and different colours present different populations. 114 genes with 10 individuals at least in each population were used. Points above the diagonal (a green line) indicate higher read counts in the Y-axis (AltHapAlignR method).

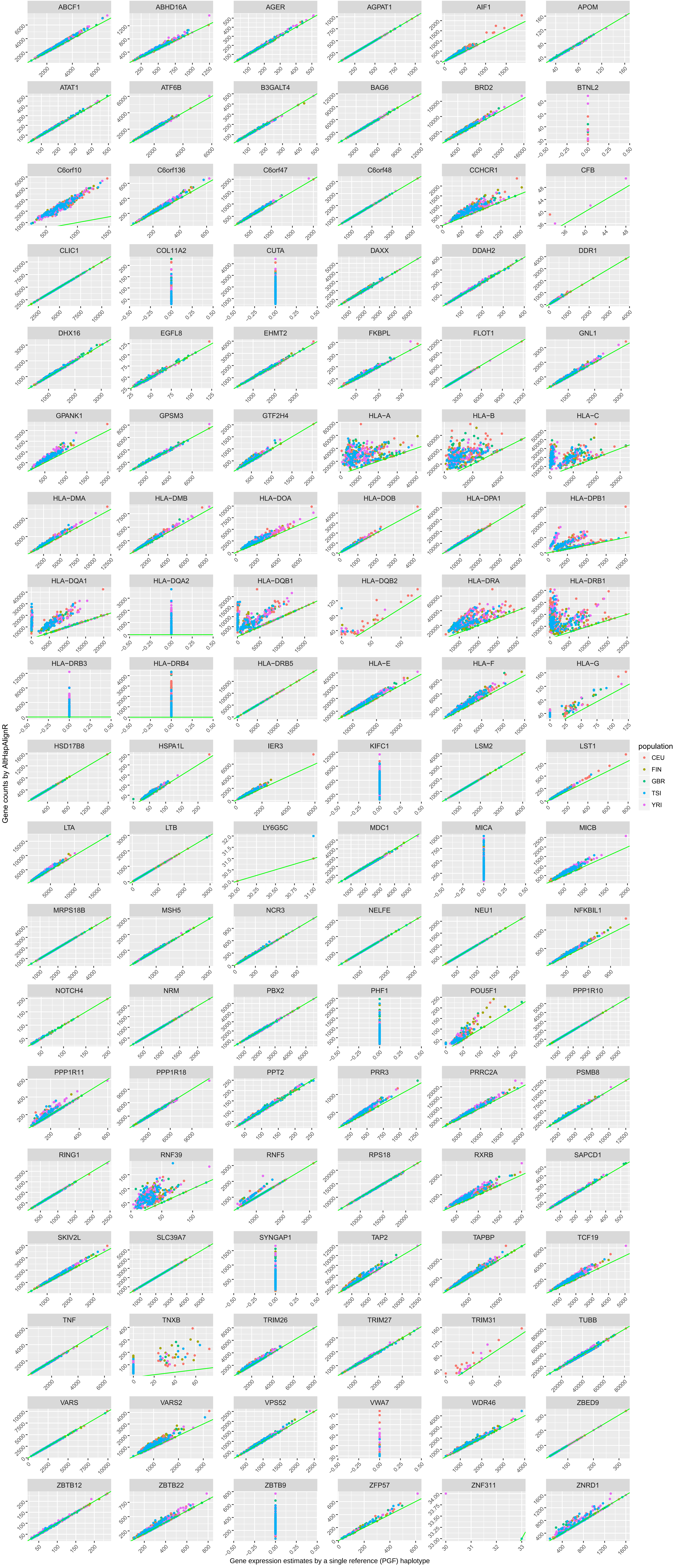
ABCF1
ABHD16A
AGER
AGPAT1
AIF1
APOM
ATAT1
ATF6B
B3GALT4
BAG6
BRD2
BTNL2
C6orf10
C6orf136
C6orf47
C6orf48
CCHCR1
CFB
CLIC1
COL11A2
CUTA
DAXX
DDAH2
DDR1
DHX16
EGFL8
EHMT2
FKBPL
FLOT1
GNL1
GPANK1
GPSM3
GTF2H4
HLA-A
HLA-B
HLA-C
HLA-DMA
HLA-DMB
HLA-DOA
HLA-DOB
HLA-DPA1
HLA-DPB1
HLA-DQA1
HLA-DQA2
HLA-DQB1
HLA-DQB2
HLA-DRA
HLA-DRB1
HLA-DRB3
HLA-DRB4
HLA-DRB5
HLA-E
HLA-F
HLA-G
HSD17B8
HSPA1L
IER3
KIFC1
LSM2
LST1
LTA
LTB
LY6G5C
MDC1
MICA
MICB
MRPS18B
MSH5
NCR3
NELFE
NEU1
NFKBIL1
NOTCH4
NRM
PBX2
PHF1
POU5F1
PPP1R10
PPP1R11
PPP1R18
PPT2
PRR3
PRRC2A
PSMB8
RING1
RNF39
RNF5
RPS18
RXRB
SAPCD1
SKIV2L
SLC39A7
SYNGAP1
TAP2
TAPBP
TCF19
TNF
TNXB
TRIM26
TRIM27
TRIM31
TUBB
VARS
VARS2
VPS52
VWA7
WDR46
ZBED9
ZBTB12
ZBTB22
ZBTB9
ZFP57
ZNF311
ZNRD1
Gene counts by AltHapAlignR
Gene expression estimates by a single reference (PGF) haplotype
population
CEU
FIN
GBR
TSI
YRI

Supplemental Figure S7. Examples of gene counts across different populations. Normalized $\log_2$ gene counts from different populations were compared with scatter plots shown. A blue line and a shaded blue are the regression line and the 95% confidence band for the mean of expression values, respectively.

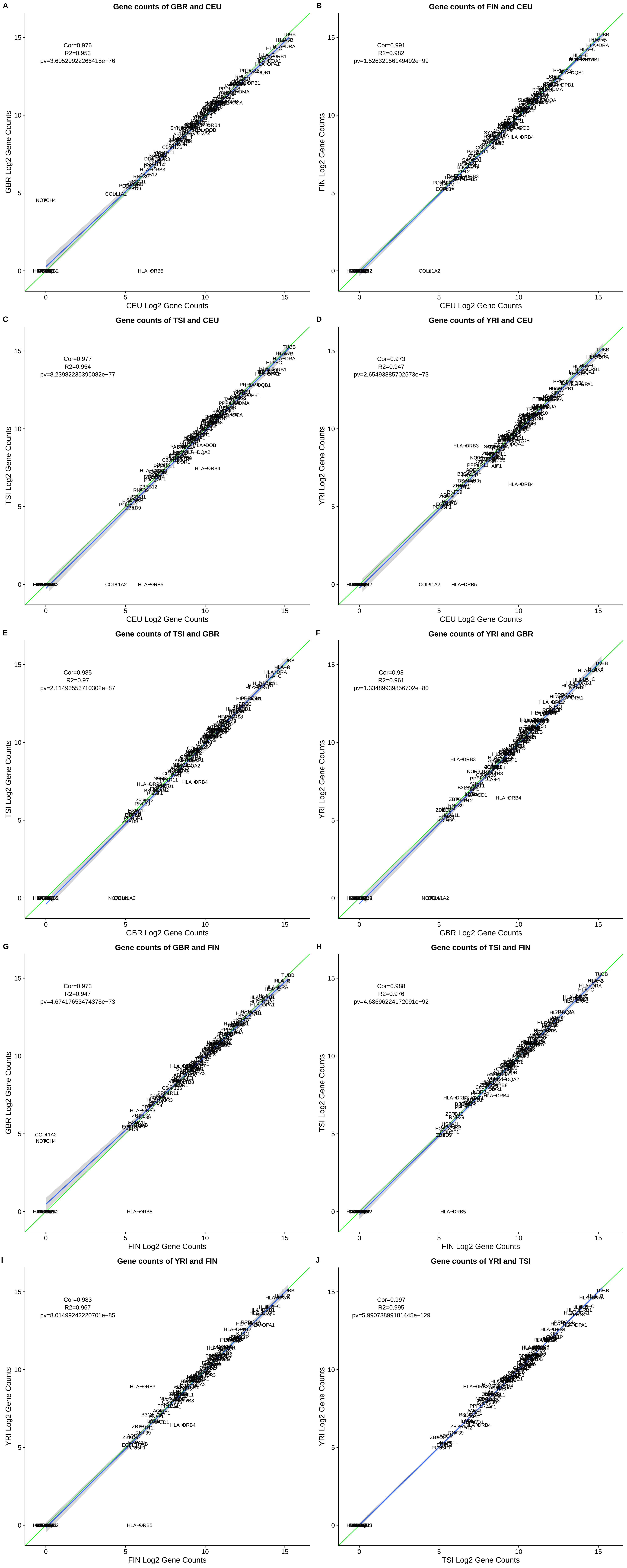
A
Gene counts of GBR and CEU
Cor=0.976
R2=0.953
pv=3.60529922266415e−76
GBR Log2 Gene Counts
CEU Log2 Gene Counts
B
Gene counts of FIN and CEU
Cor=0.991
R2=0.982
pv=1.52632156149492e−99
FIN Log2 Gene Counts
CEU Log2 Gene Counts
C
Gene counts of TSI and CEU
Cor=0.977
R2=0.954
pv=8.23982235395082e−77
TSI Log2 Gene Counts
CEU Log2 Gene Counts
D
Gene counts of YRI and CEU
Cor=0.973
R2=0.947
pv=2.65493885702573e−73
YRI Log2 Gene Counts
CEU Log2 Gene Counts
E
Gene counts of TSI and GBR
Cor=0.985
R2=0.97
pv=2.11493553710302e−87
TSI Log2 Gene Counts
GBR Log2 Gene Counts
F
Gene counts of YRI and GBR
Cor=0.98
R2=0.961
pv=1.33489939856702e−80
YRI Log2 Gene Counts
GBR Log2 Gene Counts
G
Gene counts of GBR and FIN
Cor=0.973
R2=0.947
pv=4.67417653474375e−73
GBR Log2 Gene Counts
FIN Log2 Gene Counts
H
Gene counts of TSI and FIN
Cor=0.988
R2=0.976
pv=4.68696224172091e−92
TSI Log2 Gene Counts
FIN Log2 Gene Counts
I
Gene counts of YRI and FIN
Cor=0.983
R2=0.967
pv=8.01499242220701e−85
YRI Log2 Gene Counts
FIN Log2 Gene Counts
J
Gene counts of YRI and TSI
Cor=0.997
R2=0.995
pv=5.99073899181445e−129
YRI Log2 Gene Counts
TSI Log2 Gene Counts

List of Supplemental Tables (available as individual Excel files)

Supplemental_Table_S1. List of genes excluded in this analysis.

Supplemental_Table_S2. Results of application of AltHapAlignR to analyse GEUVADIS data (HG00096.1).

Supplemental_Table_S3. Differentially expressed genes between AltHapAlignR and single reference-based mapping.

Supplemental_Table_S4. Differentially expressed genes between populations.
